# Supplementary material for: Estimating the impact of missed colorectal cancer diagnoses on life expectancy in Minamisoma City following the 2011 triple disaster
Source: PLoS One. 2025 Jun 10;20(6):e0324822. doi: 10.1371/journal.pone.0324822 (PMC12151436; doi:10.1371/journal.pone.0324822)
Supplement: S1 Table — (DOCX) [file pone.0324822.s001.docx]

S1 Table：Relative survival rates for men by age and stage of colorectal cancer

| Age group | Years after diagnosis | Stage I | Stage II | Stage III | Stage IV |
| --- | --- | --- | --- | --- | --- |
| 40-49yr | 1 year | 1 (1 - 1) | 1 (1 - 1) | 1 (1 - 1) | 0.797 (0.903 - 0.69) |
|  | 2 year | 1 (1 - 1) | 1 (1 - 1) | 0.935 (0.999 - 0.87) | 0.618 (0.747 - 0.49) |
|  | 3 year | 1 (1 - 1) | 0.957 (1 - 0.876) | 0.867 (0.954 - 0.779) | 0.413 (0.544 - 0.283) |
|  | 4 year | 1 (1 - 1) | 0.957 (1 - 0.876) | 0.867 (0.954 - 0.779) | 0.207 (0.315 - 0.1) |
|  | 5 year | 1 (1 - 1) | 0.862 (0.995 - 0.729) | 0.824 (0.923 - 0.725) | 0.104 (0.185 - 0.023) |
|  | 6 year | 1 (1 - 1) | 0.862 (0.995 - 0.729) | 0.778 (0.887 - 0.67) | 0.078 (0.15 - 0.007) |
|  | 7 year | 1 (1 - 1) | 0.817 (0.967 - 0.666) | 0.778 (0.887 - 0.67) | 0.078 (0.15 - 0.007) |
|  | 8 year | 0.972 (1 - 0.89) | 0.82 (0.971 - 0.669) | 0.778 (0.887 - 0.67) | 0.078 (0.15 - 0.007) |
|  | 9 year | 0.972 (1 - 0.89) | 0.824 (0.975 - 0.672) | 0.778 (0.887 - 0.67) | 0.078 (0.15 - 0.007) |
|  | 10 year | 0.979 (1 - 0.896) | 0.768 (0.937 - 0.598) | 0.789 (0.9 - 0.679) | 0.078 (0.15 - 0.007) |
| 50-59yr | 1 year | 1 (1 - 1) | 0.991 (1 - 0.966) | 0.943 (0.981 - 0.905) | 0.759 (0.825 - 0.692) |
|  | 2 year | 1 (1 - 0.987) | 0.967 (1 - 0.924) | 0.913 (0.96 - 0.866) | 0.533 (0.611 - 0.455) |
|  | 3 year | 0.995 (1 - 0.972) | 0.967 (1 - 0.924) | 0.883 (0.937 - 0.829) | 0.375 (0.451 - 0.299) |
|  | 4 year | 0.986 (1 - 0.956) | 0.966 (1 - 0.916) | 0.862 (0.92 - 0.803) | 0.27 (0.34 - 0.2) |
|  | 5 year | 0.968 (1 - 0.93) | 0.942 (1 - 0.882) | 0.85 (0.912 - 0.788) | 0.2 (0.263 - 0.137) |
|  | 6 year | 0.951 (0.995 - 0.906) | 0.918 (0.987 - 0.849) | 0.829 (0.894 - 0.763) | 0.174 (0.234 - 0.114) |
|  | 7 year | 0.951 (0.995 - 0.906) | 0.918 (0.987 - 0.849) | 0.808 (0.877 - 0.739) | 0.166 (0.226 - 0.107) |
|  | 8 year | 0.951 (0.995 - 0.906) | 0.918 (0.987 - 0.849) | 0.806 (0.876 - 0.735) | 0.149 (0.206 - 0.092) |
|  | 9 year | 0.969 (1 - 0.922) | 0.918 (0.987 - 0.849) | 0.804 (0.876 - 0.732) | 0.141 (0.197 - 0.085) |
|  | 10 year | 0.962 (1 - 0.911) | 0.923 (1 - 0.843) | 0.793 (0.868 - 0.718) | 0.133 (0.188 - 0.078) |
| 60-69yr | 1 year | 0.99 (1 - 0.971) | 0.986 (1 - 0.96) | 0.961 (0.989 - 0.933) | 0.761 (0.821 - 0.701) |
|  | 2 year | 0.99 (1 - 0.971) | 0.971 (1 - 0.936) | 0.916 (0.955 - 0.876) | 0.488 (0.558 - 0.418) |
|  | 3 year | 0.995 (1 - 0.968) | 0.948 (0.994 - 0.902) | 0.887 (0.933 - 0.841) | 0.337 (0.404 - 0.271) |
|  | 4 year | 0.992 (1 - 0.96) | 0.944 (0.994 - 0.894) | 0.865 (0.916 - 0.814) | 0.255 (0.317 - 0.194) |
|  | 5 year | 0.985 (1 - 0.948) | 0.941 (0.996 - 0.887) | 0.861 (0.915 - 0.808) | 0.2 (0.257 - 0.143) |
|  | 6 year | 0.966 (1 - 0.921) | 0.929 (0.99 - 0.869) | 0.853 (0.909 - 0.796) | 0.188 (0.244 - 0.131) |
|  | 7 year | 0.953 (1 - 0.903) | 0.936 (0.999 - 0.873) | 0.844 (0.904 - 0.784) | 0.175 (0.231 - 0.119) |
|  | 8 year | 0.954 (1 - 0.901) | 0.934 (1 - 0.867) | 0.834 (0.897 - 0.771) | 0.175 (0.231 - 0.119) |
|  | 9 year | 0.942 (1 - 0.884) | 0.934 (1 - 0.862) | 0.84 (0.905 - 0.775) | 0.165 (0.222 - 0.109) |
|  | 10 year | 0.9 (0.964 - 0.836) | 0.912 (0.99 - 0.834) | 0.818 (0.887 - 0.748) | 0.152 (0.207 - 0.097) |
| 70-79yr | 1 year | 1 (1 - 0.98) | 0.976 (1 - 0.943) | 0.987 (1 - 0.957) | 0.712 (0.785 - 0.638) |
|  | 2 year | 1 (1 - 0.975) | 0.97 (1 - 0.925) | 0.959 (1 - 0.914) | 0.431 (0.512 - 0.351) |
|  | 3 year | 1 (1 - 0.962) | 0.928 (0.986 - 0.87) | 0.971 (1 - 0.92) | 0.291 (0.366 - 0.216) |
|  | 4 year | 1 (1 - 0.957) | 0.939 (1 - 0.875) | 0.962 (1 - 0.902) | 0.222 (0.292 - 0.152) |
|  | 5 year | 1 (1 - 0.951) | 0.919 (0.993 - 0.846) | 0.857 (0.934 - 0.781) | 0.189 (0.256 - 0.122) |
|  | 6 year | 1 (1 - 0.943) | 0.937 (1 - 0.857) | 0.844 (0.927 - 0.76) | 0.163 (0.228 - 0.099) |
|  | 7 year | 1 (1 - 0.934) | 0.948 (1 - 0.861) | 0.847 (0.937 - 0.758) | 0.138 (0.2 - 0.076) |
|  | 8 year | 1 (1 - 0.924) | 0.92 (1 - 0.823) | 0.819 (0.916 - 0.723) | 0.138 (0.2 - 0.076) |
|  | 9 year | 1 (1 - 0.916) | 0.932 (1 - 0.827) | 0.823 (0.926 - 0.72) | 0.138 (0.2 - 0.076) |
|  | 10 year | 1 (1 - 0.91) | 0.926 (1 - 0.813) | 0.809 (0.919 - 0.698) | 0.148 (0.218 - 0.078) |

Values represent relative survival rates and 95% confidence intervals.
