# Supplementary material for: Estimating the impact of missed colorectal cancer diagnoses on life expectancy in Minamisoma City following the 2011 triple disaster
Source: PLoS One. 2025 Jun 10;20(6):e0324822. doi: 10.1371/journal.pone.0324822 (PMC12151436; doi:10.1371/journal.pone.0324822)
Supplement: S2 Table — (DOCX) [file pone.0324822.s002.docx]

S2 Table： Relative survival rates for women by age and stage of colorectal cancer

| Age group | Yeas after diagnosis | Stage I | Stage II | Stage III | Stage IV |
| --- | --- | --- | --- | --- | --- |
| 40-49yr | 1 year | 1 (1 - 1) | 0.918 (1 - 0.786) | 0.93 (1 - 0.85) | 0.774 (0.921 - 0.627) |
|  | 2 year | 0.967 (1 - 0.909) | 0.918 (1 - 0.786) | 0.859 (0.968 - 0.75) | 0.547 (0.722 - 0.372) |
|  | 3 year | 0.967 (1 - 0.909) | 0.918 (1 - 0.786) | 0.825 (0.944 - 0.705) | 0.456 (0.632 - 0.281) |
|  | 4 year | 0.967 (1 - 0.909) | 0.918 (1 - 0.786) | 0.682 (0.828 - 0.536) | 0.274 (0.431 - 0.117) |
|  | 5 year | 0.935 (1 - 0.855) | 0.918 (1 - 0.786) | 0.648 (0.798 - 0.497) | 0.274 (0.431 - 0.117) |
|  | 6 year | 0.935 (1 - 0.855) | 0.918 (1 - 0.786) | 0.648 (0.798 - 0.497) | 0.229 (0.378 - 0.081) |
|  | 7 year | 0.935 (1 - 0.855) | 0.918 (1 - 0.786) | 0.542 (0.699 - 0.385) | 0.229 (0.378 - 0.081) |
|  | 8 year | 0.935 (1 - 0.855) | 0.918 (1 - 0.786) | 0.507 (0.664 - 0.349) | 0.184 (0.321 - 0.047) |
|  | 9 year | 0.935 (1 - 0.855) | 0.918 (1 - 0.786) | 0.507 (0.664 - 0.349) | 0.184 (0.321 - 0.047) |
|  | 10 year | 0.908 (1 - 0.81) | 0.918 (1 - 0.786) | 0.507 (0.664 - 0.349) | 0.184 (0.321 - 0.047) |
| 50-59yr | 1 year | 1 (1 - 1) | 0.984 (1 - 0.954) | 0.993 (1 - 0.977) | 0.767 (0.855 - 0.679) |
|  | 2 year | 1 (1 - 1) | 0.984 (1 - 0.954) | 0.947 (0.985 - 0.91) | 0.497 (0.601 - 0.393) |
|  | 3 year | 0.996 (1 - 0.976) | 0.952 (1 - 0.901) | 0.882 (0.936 - 0.828) | 0.45 (0.554 - 0.346) |
|  | 4 year | 0.987 (1 - 0.959) | 0.937 (0.996 - 0.877) | 0.807 (0.873 - 0.742) | 0.387 (0.489 - 0.285) |
|  | 5 year | 0.987 (1 - 0.959) | 0.937 (0.996 - 0.877) | 0.791 (0.859 - 0.723) | 0.307 (0.404 - 0.211) |
|  | 6 year | 0.987 (1 - 0.959) | 0.887 (0.963 - 0.81) | 0.774 (0.844 - 0.703) | 0.243 (0.333 - 0.153) |
|  | 7 year | 0.987 (1 - 0.959) | 0.853 (0.938 - 0.767) | 0.757 (0.829 - 0.684) | 0.228 (0.316 - 0.14) |
|  | 8 year | 0.987 (1 - 0.959) | 0.853 (0.938 - 0.767) | 0.72 (0.796 - 0.644) | 0.18 (0.261 - 0.099) |
|  | 9 year | 0.987 (1 - 0.959) | 0.853 (0.938 - 0.767) | 0.703 (0.78 - 0.625) | 0.164 (0.242 - 0.086) |
|  | 10 year | 0.998 (1 - 0.963) | 0.853 (0.938 - 0.767) | 0.686 (0.765 - 0.607) | 0.132 (0.204 - 0.06) |
| 60-69yr | 1 year | 1 (1 - 1) | 0.977 (1 - 0.945) | 0.979 (1 - 0.955) | 0.768 (0.84 - 0.695) |
|  | 2 year | 0.993 (1 - 0.972) | 0.968 (1 - 0.929) | 0.924 (0.967 - 0.881) | 0.435 (0.52 - 0.349) |
|  | 3 year | 0.993 (1 - 0.972) | 0.931 (0.986 - 0.876) | 0.904 (0.952 - 0.855) | 0.317 (0.397 - 0.237) |
|  | 4 year | 0.996 (1 - 0.971) | 0.894 (0.961 - 0.828) | 0.866 (0.922 - 0.81) | 0.286 (0.364 - 0.208) |
|  | 5 year | 0.976 (1 - 0.94) | 0.858 (0.933 - 0.782) | 0.846 (0.906 - 0.785) | 0.178 (0.244 - 0.111) |
|  | 6 year | 0.976 (1 - 0.94) | 0.85 (0.929 - 0.772) | 0.825 (0.889 - 0.762) | 0.112 (0.167 - 0.057) |
|  | 7 year | 0.963 (1 - 0.92) | 0.85 (0.929 - 0.772) | 0.823 (0.888 - 0.758) | 0.091 (0.141 - 0.04) |
|  | 8 year | 0.953 (1 - 0.904) | 0.835 (0.92 - 0.75) | 0.822 (0.888 - 0.755) | 0.091 (0.141 - 0.04) |
|  | 9 year | 0.952 (1 - 0.901) | 0.835 (0.92 - 0.75) | 0.811 (0.88 - 0.742) | 0.081 (0.129 - 0.033) |
|  | 10 year | 0.952 (1 - 0.901) | 0.853 (0.94 - 0.766) | 0.782 (0.855 - 0.709) | 0.07 (0.116 - 0.025) |
| 70-79yr | 1 year | 1 (1 - 1) | 0.963 (1 - 0.922) | 0.959 (0.995 - 0.923) | 0.647 (0.752 - 0.542) |
|  | 2 year | 1 (1 - 0.977) | 0.965 (1 - 0.919) | 0.908 (0.96 - 0.856) | 0.371 (0.478 - 0.265) |
|  | 3 year | 1 (1 - 0.973) | 0.902 (0.968 - 0.836) | 0.876 (0.937 - 0.815) | 0.233 (0.326 - 0.139) |
|  | 4 year | 1 (1 - 0.966) | 0.852 (0.931 - 0.774) | 0.826 (0.896 - 0.756) | 0.146 (0.225 - 0.067) |
|  | 5 year | 1 (1 - 0.954) | 0.858 (0.939 - 0.776) | 0.805 (0.881 - 0.729) | 0.146 (0.225 - 0.067) |
|  | 6 year | 1 (1 - 0.949) | 0.85 (0.938 - 0.763) | 0.772 (0.854 - 0.69) | 0.114 (0.187 - 0.042) |
|  | 7 year | 1 (1 - 0.942) | 0.859 (0.95 - 0.768) | 0.749 (0.836 - 0.662) | 0.114 (0.187 - 0.042) |
|  | 8 year | 1 (1 - 0.937) | 0.859 (0.95 - 0.768) | 0.739 (0.83 - 0.647) | 0.114 (0.187 - 0.042) |
|  | 9 year | 1 (1 - 0.93) | 0.880 (0.98 - 0.780) | 0.677 (0.774 - 0.58) | 0.114 (0.187 - 0.042) |
|  | 10 year | 1 (1 - 0.921) | 0.896 (1 - 0.791) | 0.68 (0.781 - 0.578) | 0.114 (0.187 - 0.042) |

Values represent relative survival rates and 95% confidence intervals.
