# Supplementary material for: Estimating the impact of missed colorectal cancer diagnoses on life expectancy in Minamisoma City following the 2011 triple disaster
Source: PLoS One. 2025 Jun 10;20(6):e0324822. doi: 10.1371/journal.pone.0324822 (PMC12151436; doi:10.1371/journal.pone.0324822)
Supplement: S3 Table — (DOCX) [file pone.0324822.s003.docx]

S3 Table Distribution of colorectal cancers detected by colorectal cancer mass screening in Japan by stage

|  | Male |  |  |  |  | Female |  |  |  |  |
| --- | --- | --- | --- | --- | --- | --- | --- | --- | --- | --- |
|  | Age groups | | | | | Age groups | | | | |
| Stage | 40-49yr | 50-59yr | 60-69yr | 70-79yr | 80yr≧ | 40-49yr | 50-59yr | 60-69yr | 70-79yr | 80yr≧ |
| I | 0.12660523 | 0.35673083 | 0.89880489 | 1.19416242 | 1.27120479 | 0.11982917 | 0.21763891 | 0.41823185 | 0.64447908 | 0.77546683 |
| II | 0.04340751 | 0.15621546 | 0.37210522 | 0.48351209 | 0.69027787 | 0.04108429 | 0.09530593 | 0.17314799 | 0.26094727 | 0.42108682 |
| III | 0.06149397 | 0.20751009 | 0.37570044 | 0.48351209 | 0.51258258 | 0.05820274 | 0.12660041 | 0.17482091 | 0.26094727 | 0.31268824 |
| IV | 0.01446917 | 0.02098417 | 0.04673785 | 0.04947566 | 0.04100661 | 0.01369476 | 0.01280229 | 0.02174806 | 0.02670158 | 0.02501506 |
| Total | 0.24597587 | 0.74144056 | 1.69334841 | 2.21066225 | 2.51507184 | 0.23281095 | 0.45234754 | 0.78794881 | 1.19307521 | 1.53425695 |

units; cases/1000 persons

yr: years
