# Supplementary material for: Estimating the impact of missed colorectal cancer diagnoses on life expectancy in Minamisoma City following the 2011 triple disaster
Source: PLoS One. 2025 Jun 10;20(6):e0324822. doi: 10.1371/journal.pone.0324822 (PMC12151436; doi:10.1371/journal.pone.0324822)
Supplement: S1 Fig — (DOCX) [file pone.0324822.s004.docx]

S1 Figure Additional loss of life expectancy resulting from changes in colorectal cancer stage due to a 1-year diagnostic delay per 10000 persons


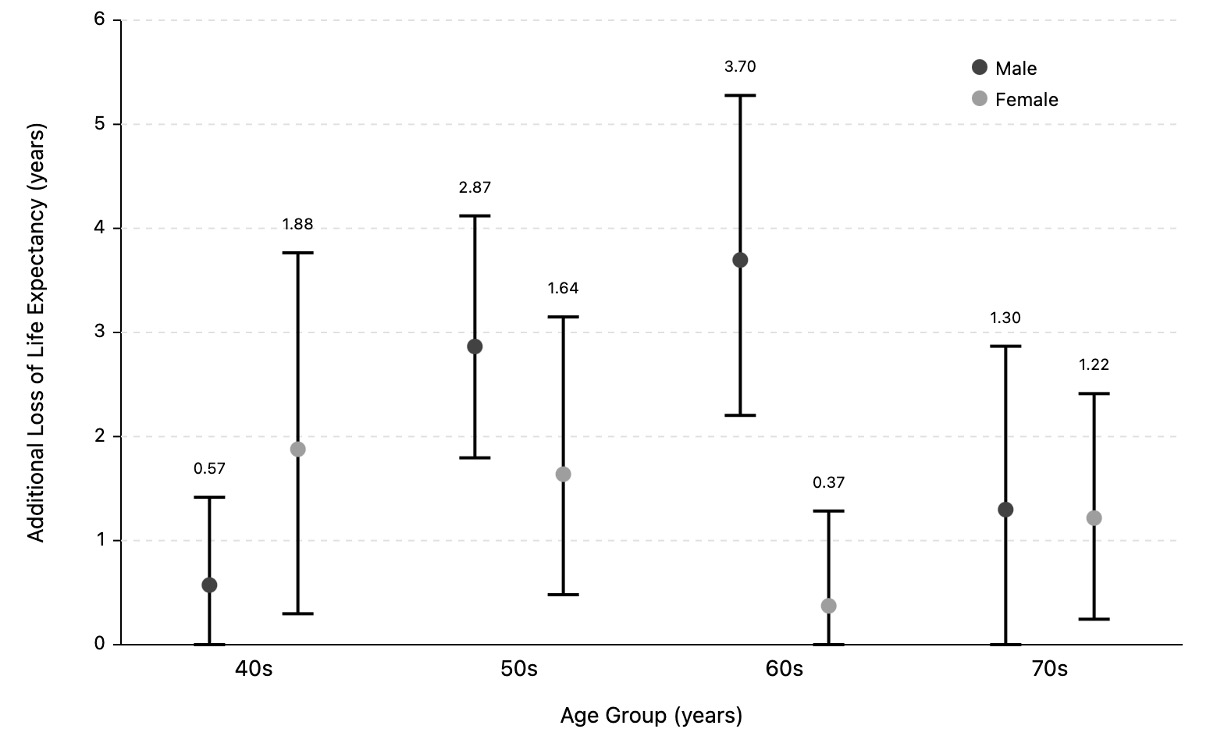


*There are point estimates (dots) with 95% uncertainty intervals (vertical lines)
