# Supplementary material for: Estimating the impact of missed colorectal cancer diagnoses on life expectancy in Minamisoma City following the 2011 triple disaster
Source: PLoS One. 2025 Jun 10;20(6):e0324822. doi: 10.1371/journal.pone.0324822 (PMC12151436; doi:10.1371/journal.pone.0324822)
Supplement: Attachment — Submitted filename: PlosOne Comment.pdf [file pone.0324822.s006.pdf]

Overall representation is good, the novelty, description and paper sections are managed well.

Only few things need to be considered to make it more valuable:

- Lack of Crystal Ball software Tool configuration
- Also include the limitations of Estimated shift of CRC stage in case of reduced screening.
- The visualization is not enough (recommendations: Bar Plotting OR Spider Plotting)
