# Supplementary material for: Estimating the impact of missed colorectal cancer diagnoses on life expectancy in Minamisoma City following the 2011 triple disaster
Source: PLoS One. 2025 Jun 10;20(6):e0324822. doi: 10.1371/journal.pone.0324822 (PMC12151436; doi:10.1371/journal.pone.0324822)
Supplement: Attachment — Submitted filename: Response to the comments.docx [file pone.0324822.s007.docx]

**Response to the comments**

Reviewers' comments:

**Reviewer #1:** Your manuscript provides a valuable contribution to the field of disaster medicine and public health. The modeling approach and analysis are rigorous, and the policy implications of your findings are clear. To further strengthen your paper, consider addressing the points mentioned in the editor's recommendation, particularly expanding on the limitations of your assumptions and the potential impact of missed screenings on precancerous lesions.

**Response**

We appreciate your comments. We have modified the manuscript according to the editor’s comments and reviewer’s comments.

**Comments**
I recommend accepting the manuscript with minor revisions, particularly focusing on the following areas:
1. Enhance the discussion of limitations: Expand on the limitations related to the assumptions made in the LLE calculations and the generalizability of the findings to other populations.

**Response**

Thank you for this helpful comment. We have added to the limitations section a discussion of our assumption that the proportion of colorectal cancers detected through screening in our study population would match national statistics. We acknowledge that this assumption may not hold true for other populations. To address the generalizability of our findings, we further elaborated on the need to consider key process indicators related to colorectal cancer screening, such as population risk, primary screening positive rate, and secondary screening attendance rate. When applying our methodology to other populations, these factors should be carefully evaluated and adjustments made as needed to ensure the validity of the results. We have added the following sentences.

Page35 Lines109-120

This study has several limitations. First, in calculating LLE, we assumed that the proportion of CRC cases detected through the fecal occult blood method in the study population would match the national statistics for Japan. This assumption implies that if the population risk is equivalent, and the primary screening positive rate and secondary screening attendance rate are stable, the number of CRC diagnoses can be predicted for any population undergoing the screening. However, this may not hold true for other populations. Notably, Japan has a known low colonoscopy screening attendance rate, which could be higher in populations with better educational approaches to CRC screening. When considering the generalizability of this research method to other populations, it is crucial to evaluate whether the population's CRC risk and indicators for each screening process are comparable to this study. Adjusting these factors as needed may enhance the generalizability of this study.

**Comments**
2. Clarify the impact of screening on precancerous lesions: Address the potential impact of missed CRC screenings on the detection and removal of precancerous lesions, which could further influence the study's conclusions.

**Response**

Thank you for your valuable comment. As you pointed out, one of the limitations of this study is that it does not include the therapeutic effect of precancerous lesions as a cancer prevention effect. We have added further details regarding this point to the limitations section. In this model, we calculated the effect on the LEE of detected colorectal cancer over a 10-year span. On the other hand, it is generally considered that it takes at least 10-15 years for an adenoma to lead to colorectal cancer death, so we believe it is acceptable not to include it in this model. Certainly, a model that measures the long-term effects of these treatments is needed, and we have indicated the need for further research.

Page35-6 Lines121-132

Second, this study does not account for the effect of identifying and excising colorectal polyps, which are precancerous CRC lesions. The impact of CRC screening encompasses both early CRC detection to suppress cancer progression and the prevention of CRC development by detecting and removing precancerous lesions. Our estimations only include the effect of reduced life expectancy due to CRC detected through screening and do not consider the preventive effect of identifying and treating precancerous lesions. Consequently, the increase in LLE due to the decline in CRC screening participation may be underestimated. However, the time from advanced adenoma to CRC death is estimated to be 10-15 years [31], suggesting a negligible impact within our model's timeframe. To estimate the effect of LLE, including the treatment effect of precancerous lesions, a model that evaluates long-term impacts such as microsimulation models [32], needs to be constructed.

**Comments**
3. Include a brief mention of limitations in the abstract: Add a sentence in the abstract that briefly acknowledges the study's limitations.

**Response**

We appreciate your comment. We have modified the abstract as follows.

Page 37 Lines 149-159

**Conclusions:** Although these data could be used to determine health policy goals following a catastrophic disaster, they should be interpreted with caution because they may underestimate the impact of CRC screening. This study has limitations, including the assumption of uniform national CRC screening and treatment performance and the exclusion of the preventive effect of polyp removal.
