# Supplementary material for: Estimating the impact of missed colorectal cancer diagnoses on life expectancy in Minamisoma City following the 2011 triple disaster
Source: PLoS One. 2025 Jun 10;20(6):e0324822. doi: 10.1371/journal.pone.0324822 (PMC12151436; doi:10.1371/journal.pone.0324822)
Supplement: Attachment — Submitted filename: Response letter.docx [file pone.0324822.s008.docx]

Journal Requirements:

Please review your reference list to ensure that it is complete and correct. If you have cited papers that have been retracted, please include the rationale for doing so in the manuscript text, or remove these references and replace them with relevant current references. Any changes to the reference list should be mentioned in the rebuttal letter that accompanies your revised manuscript. If you need to cite a retracted article, indicate the article’s retracted status in the References list and also include a citation and full reference for the retraction notice.

**Reply**

For the following cited papers, minor corrections to the terminology description in the abstract have been declared, but not retraceted. We therefore cited the article.

Satoh H, Ohira T, Hosoya M, Sakai A, Watanabe T, Ohtsuru A, et al. Evacuation after the Fukushima Daiichi Nuclear Power Plant Accident Is a Cause of Diabetes: Results from the Fukushima Health Management Survey. J Diabetes Res. 2015;2015:627390. Epub 2015/06/25. doi: 10.1155/2015/627390. PubMed PMID: 26106625; PubMed Central PMCID: PMCPMC4461763.

**Comments**

Reviewer #2: Overall representation is good, the novelty, description and paper sections are managed well.

Only few things need to be considered to make it more valuable:

• Lack of Crystal Ball software Tool configuration

**Reply**

Thank you for your comment. We have amended the description of the Monte Carlo simulation with Crystal Ball as follows.

Page 19 line 325-338

To estimate uncertainty, Monte Carlo simulation was performed using Crystal Ball software (Oracle, Redwood City, CA, USA). For screening participation rates in Minamisoma City and national colorectal cancer detection rates, we calculated standard errors and defined probability distributions. For 10-year relative mortality rates of colorectal cancer, we utilized the reported 95% confidence intervals (S1, S2 tables). The simulation was conducted with 10,000 iterations for each age group and sex category. For each iteration, the model randomly sampled values from the defined probability distributions for all input parameters and calculated the resulting outcomes (number of missed CRC cases, stage distributions, and associated LLEs). The 95% uncertainty intervals (UIs) reported throughout our results represent the 2.5th and 97.5th percentiles of the output distributions from these 10,000 iterations. For key outputs such as the number of missed CRC diagnoses, the additional LLE due to stage progression, and the cost per life-year saved (CPLYS), we reported both the point estimates (based on the mean of the simulation results) and the 95% UIs.

**Comments**

• Also include the limitations of Estimated shift of CRC stage in case of reduced screening.

**Reply**

Thank you for your suggestion. We have added this issue in the limitation as follows.

Page 37, line 143-150

Fourth, information regarding the progression of colorectal cancer stages due to a one-year delay in diagnosis is based on limited literature. The assumption that 10% of early-stage (I and II) colorectal cancers would progress to more advanced stages within one year may not fully capture the complex nature of cancer progression, which can vary significantly between individuals and cancer subtypes. Future research with more robust data on stage progression rates specific to the Japanese population would strengthen our estimation.

**Comments**

• The visualization is not enough (recommendations: Bar Plotting OR Spider Plotting)

**Reply**

In addition to the Table, additional loss of life expectancy resulting from changes in colorectal cancer stage due to a 1-year diagnostic delay has been added to the S1 figure.

**Comments**

Reviewer #3: Very nice and methodologically sound and transparent paper on missed cancer cases in screening program due to a national disaster. I recommend it for publication.

**Reply**

We appreciate your review and comments.

**Comments**

Reviewer #4: I don’t like the abstract I will look review detail if you give go ahead (no response)

Importsnt topic very much in my field. I was a silent academic interacting w the interested screeners

I don’t like the abstract

However ,Yes, I appreciate and very much sympathize w the authors cultural requirements

The abstract is 1.overloaded details re methods Results

**Reply**

We appreciate your comments. We have modified the abstract as follows.

**Background:** After the 2011 Great East Japan Earthquake, participation in colorectal cancer (CRC) screening significantly decreased in Minamisoma City, Fukushima Prefecture. However, the long-term health effects of this decline in screening participation have not been quantified. This study aims to construct a model to evaluate the impact of post-disaster decreases in CRC screening participation on population health.

**Methods:** We utilized the population and CRC screening data targeting 40-74 years-old residents in Minamisoma City. We compared the actual screening participation in 2011 with projected participation rates based on pre-disaster levels to estimate the number of residents who missed screening due to the disaster. Based on national CRC screening performance data and stage-specific survival rates in Japan, we estimated the number of missed CRC cases and modeled the additional the loss of life expectancy (LLE) due to CRC resulting from a one-year delay in diagnosis.

**Res**ults: The estimated number of colorectal cancer cases that might have been missed due to decreased screening participation was 1.794 (95% uncertainty interval: 1.597 to 1.994) for men and 1.203 (0.931 to 1.491) for women. The missed detection opportunities estimated result in 0.428 (0.282 to 0.582) person-years [2.684 (1.793 to 3.604) years per 10,000 persons] and 0.229 (0.103 to 0.372) person-years [0.993 (0.450 to 1.608) years per 10,000 persons] of additional LLE for men and women, respectively. The estimated cost per life-year saved was $1.12\times{10}^{6} (0.81\times{10}^{6} to 1.62\times{10}^{6} )$yen for men and $3.65\times{10}^{6} (2.02\times{10}^{6} to 7.19\times{10}^{6} )$ yen for women, respectively.

**Conclusions:** The calculated additional LLE due to missed CRC screening was relatively small but suggests preventive health services should be considered in disaster response planning. These findings provide a quantitative framework for evaluating health impacts of service disruptions.

**Comments**

2. This will be dangerously lost (misled)on reader uncertain sbout colonoscopy in their practices (expert opinion public heslth actor needs to go to text)

**Reply**

Colonoscopy is generally recommended in all cases of positive feacal immunochemical test (FIT). The administration repeatedly contacts and recommends those who have not undergone it to undergo colonoscopy. In this estimation, the impact is estimated by using statistics from Japan on the number of colorectal cancers diagnosed from people who have undergone the FIT. The average colonoscopy compliance rate and the percentage of cancers detected in the process are factored in. The following has been added to the method regarding the usual response.

Page 9 Line 142-144

Colorectal cancer screening data of Minamisoma is compiled for those who participated in the annually recommended faecal immunochemical test (FIT). Colonoscopy is recommended if FIT is positive.

3.Please it needs lacks clear statements- tech jargon is not for abstract section

A)how many cancers were discovered at later staged updtsged (what stage 4,3,2 /10000 adults

**Reply**

The estimated missed cancers in relation to men and women are listed in the abstract as follows. Detailed estimates of the number of missed cancers per stage have been omitted from the abstract, as they are listed in table 3.

**Abstract**

**Methods:** We utilized the population and CRC screening data targeting 40-74 years-old residents in Minamisoma City. We compared the actual screening participation in 2011 with projected participation rates based on pre-disaster levels to estimate the number of residents who missed screening due to the disaster. Based on national CRC screening performance data and stage-specific survival rates in Japan, we estimated the number of missed CRC cases and modeled the additional the loss of life expectancy (LLE) due to CRC resulting from a one-year delay in diagnosis.

B)which population is target for extra intervention (young or elderly define age cut

**Reply**

We appreciate your comments. We have added the eli

Page 9 Line 139-140

We focused on individuals aged 40–74 years who were eligible for municipal CRC screening in Minamisoma in 2011.

C)How many otherwise avoidable deaths !!

**Reply**

The extent to which death occurs is not calculated in this simulation. In this case, life expectancy lost is calculated as an assessment of the risk caused by not undergoing screening. Although there are methods to calculate lost life expectancy from additional deaths that occur, we believe that these are not optimal when estimating the short-term impact in a small population such as in this case.

D)Is this enough evidence health loss , to support which/ sny salvage intervention ?

Whose (why do) criteria support a special intervention ?

**Reply**

We appreciate your important question. There are no clear criteria on whether intervention should be undertaken, and efforts should be made by disaster-affected areas to regain the effectiveness of their peacetime screening programmes compared to the priorities of other issues. As we describe in our discussion, the results of this simulation can be used for comparison with other health effects that have occurred since the disaster. From the perspective of lost life expectancy, we can conclude that the administrative priority should be on diabetes control, as the burden of diabetes is higher compared to the increase in diabetes that occurred after the disaster. On the other hand, from the CPLYS perspective, measures to increase participation in colorectal cancer screening are cost-effective, so it is reasonable to take measures to increase the declining participation rate. We should explore the desired intervention method, but it would be desirable to consider implementing a reintroduction of screening awareness and enhanced follow-up programmes to restore screening participation rates. We have added this issue as follows.

Page 31 Line 31-33

The desired intervention is a subject for future research, but it would be desirable to consider implementing a reintroduction of screening awareness and enhanced follow-up programmes to restore screening participation rates.

E) did they adjust for real - world routine non compliance

**Reply**

This is a important topic. In colorectal cancer screening, participation rates vary by region. The genelalizabitity in this regard is described in the limitaion below.

**Page 38 Line 150-151**

Finally, the Minamisoma City population was < 70,000 at the time of the disaster, which is a relatively small target population. This was compounded by the fact that the CRC screening participation rate was low (in the 10% range) before the disaster, suggesting a potentially limited number of study participants. These factors introduced significant limitations during the verification process. However, to mitigate these limitations, we employed Monte Carlo analysis to enhance the validity of our research.

**Comments**

If they are concerned that they underestimated the impsct this is a very concerning message ( this is a point for limitations not abstract )

Do They by extension dangerously downplay screening

( I don’t like that To msny doubt provokers come across my desk! History changing tech makes them wrong advice going forward

**Reply**

Our interpretation is also that the decline in colorectal cancer screening after the disaster should not be underestimated. We do not underestimate the impact of screening. We are only adding a note that the impact shown may be a numerical underestimation. In other words, the actual impact of the drop in screening uptake may be greater than that simulated here, and may require more vigilance. This is a Simulation study and there are usual limitations to the estimates.
